# Whole-genome sequencing reveals novel genes in ossification of the posterior longitudinal ligament of the thoracic spine in the Chinese population

**DOI:** 10.1186/s13018-018-1022-8

**Published:** 2018-12-22

**Authors:** Chen Liang, Peng Wang, Xiao Liu, Chenlong Yang, Yunlong Ma, Lei Yong, Bin Zhu, Xiaoguang Liu, Zhongjun Liu

**Affiliations:** 1 Department of Orthopaedics, Peking University Third Hospital, No. 49, North Garden Road, Haidian District, Beijing, 100191 China; 2 The Centre for Pain Medicine, Peking University Third Hospital, No. 49, North Garden Road, Haidian District, Beijing, 100191 China

**Keywords:** Ossification of the posterior longitudinal ligament, Thoracic, Whole-genome sequencing, Susceptible gene

## Abstract

**Background:**

Ossification of the posterior longitudinal ligament (OPLL) of the spine is a complex, multifactorial disease. Although several genes that are linked to cervical OPLL susceptibility have been reported, specific genetic studies regarding thoracic OPLL are lacking. Whole-genome sequencing has been considered as an efficient strategy to search for disease-causing genes.

**Methods:**

We analysed whole-genome sequences in a cohort of 25 unrelated patients with thoracic OPLL. Bioinformatics analysis and various algorithms were used to predict deleterious variants. Sanger sequencing was used to confirm the variants.

**Results:**

Four deleterious mutations in three genes (c.2716C>T (p.Arg906Cys) in collagen type VI α6 (*COL6A6*); c.1946G>C (p.Gly649Ala) in collagen type IX α1 (*COL9A1*); and c.301T>C (p.Ser101Pro) and c.171A>G (p.Ile57Met) in toll-like receptor 1 (*TLR1*)) were successfully identified. All the variants were confirmed by Sanger sequencing.

**Conclusion:**

The novel deleterious mutations of the three genes may contribute to the development of thoracic OPLL.

## Introduction

Ossification of the posterior longitudinal ligament (OPLL) of the spine is characterized by haeterotopic bone formation in cervical or thoracic ligaments, causing myelopathy, and OPLL is most prevalent in the East Asian population [1, 2]. As thoracic OPLL (T-OPLL) progresses latently, patients may be underdiagnosed until the advanced stages of the disease when the spinal cord is severely compressed by ectopic ossification. In addition, the surgical treatment of T-OPLL is challenging due to the complicated anatomical structures, and T-OPLL generally has a less favourable prognosis than its cervical counterpart [3, 4]. A comprehensive understanding of the pathogenesis of T-OPLL may provide new insight into the therapeutic approaches for T-OPLL.

Though the aetiology of OPLL remains unclear, it has been widely accepted that this entity is a multifactorial disease influenced by numerous genetic and non-genetic factors [5]. Non-genetic factors include mechanical stress, the degeneration process, diet, and biological rhythm. Compared to the cervical spine, the thoracic spine has less range of motion as it is limited by the thorax. In addition, the thoracic spine is more stable and experiences less mechanical stress than the cervical spine. Thus, the thoracic spine is less susceptible to degeneration. Consequently, we hypothesized that genetic factors play a vital role in the aetiology and development of T-OPLL. Previous genetic studies of OPLL have revealed the following osteogenic gene loci that may be involved in the pathogenesis of cervical OPLL: *NPPS*, *COL6A1*, *COL11A2*, *BMP2*, *BMP4*, *BMP9*, *TGF-β1*, *TGF-β3*, *TGFΒR2*, *ESR1*, *FGFR1*, *IL-1β*, *IL-15RA*, *RUNX2* and *RSPO2* [6–18]. A recent study revealed two deleterious variants of *COL6A1* and *IL17RC* in patients with T-OPLL [19]. However, there is inadequate research regarding susceptibility genes for T-OPLL. Therefore, there is an urgent need to identify pathogenic genes to provide novel approaches for future investigation and intervention of T-OPLL. Whole-genome sequencing represents an effective, accurate and reproducible strategy for the identification of specific variants in individual human genomes [20, 21]. The present study analysed data from whole-genome sequencing from 25 sporadic OPLL patients to identify pathogenic genes for T-OPLL. Sanger DNA sequencing was used to validate the variants disclosed by whole-genome sequencing. Single nucleotide polymorphisms (SNPs) were analysed with bioinformatics. The present study reported several pathogenic variants in genes associated with T-OPLL in the Chinese population.

## Materials and methods

### Patients

Twenty-five unrelated Chinese patients with T-OPLL were consecutively recruited between 2012 and 2016 from the Department of Orthopaedics at Peking University Third Hospital (PUTH), including 12 male and 13 female patients with an average age of 52.4 years ranging from 29 to 70 years. The present study was conducted with the approval of the Ethics Committee of the PUTH Institutional Review Board. Written informed consent was obtained from all patients whose specimens and clinical information were used for the present study. All patients received radiological examinations, including plain radiographs, computed tomography (CT) and magnetic resonance imaging (MRI). T-OPLL was diagnosed based on clinical and radiological evidence by at least two experienced spinal surgeons. The inclusion criteria were as follows: (a) > 18 years old and (b) both CT and MRI evidence of T-OPLL were available. The exclusion criteria were as follows: (a) metabolic diseases, such as hypophosphataemic rickets, acromegaly, hyperparathyroidism, diffuse idiopathic skeletal hyperostosis and pituitary diseases; (b) administration of medications interfering with bone or calcium metabolism, such as oral contraceptives, calcium, vitamin D and glucocorticoids; (c) concomitant developmental malformations; or (d) family history of hereditary disease [22]. Neurological status and surgical outcome were assessed using the Japanese Orthopaedic Association (JOA) scoring system for thoracic myelopathy [4].

### Whole-genome sequencing

Genomic DNA was extracted from whole blood with the TIANamp Blood DNA kit (TIANGEN BIOTECH, Beijing, China). Quality and quantity of DNA were assessed with a NanoDrop 2000 spectrophotometer (Thermo Scientific, Wilmington, DE, USA). Whole-genome sequencing was performed on an Illumina HiSeq X Ten platform using the GenCap custom enrichment kit (MyGenostics, Beijing). The experiment was performed according to the manufacturer’s protocol.

### Bioinformatics analysis

Bioinformatics analysis started with the raw sequencing data from the Illumina pipeline. Raw data were processed and filtered, and low-quality reads were discarded. Burrows-Wheeler Aligner (BWA) software was used to align the clean reads to the human reference genome (GRCh37/HG19). Genome Analysis Toolkit (GATK) was applied for variant analysis, including local realignment around insertion/deletions (InDels) and base quality score recalibration. Duplicate reads were discarded using Picard. A quality control (QC) system guaranteed high-quality data throughout the entire analysis pipeline.

### Validation and evaluation of mutations

Sanger DNA sequencing was used to validate the accuracy of all identified variants. Variant frequency was compared with the 1000G SNP database (http://www.1000genomes.org/). Analysis for potential deleterious mutations was performed using various algorithms, including PolyPhen2 (http://genetics.bwh.harvard.edu/pph2/) [23], SIFT (http://sif.jcvi.org/) [24], Mutation Assessor (http://mutationassessor.org/r3/), Mutation Taster (http://www.mutationtaster.org/) [25] and GERP++ prediction (http://mendel.stanford.edu/SidowLab/downloads/gerp/) [26]. In the present study, the variant frequency was combined with at least four of the five algorithms above to prioritize potential pathogenic variants.

### Sanger sequencing

DNA was amplified using custom oligonucleotide primers. PCR amplification of all SNPs was conducted using Ex-Taq premix (Takara, Japan). The following reaction conditions were used: 96 °C for 5 min; 35 cycles at 96 °C for 20 s, 52 °C for 30 s and 72 °C for 60 s; and 72 °C for 5 min. PCR assays (total volume of 50 μL) contained 0.25 μL of Ex-Taq premix (5 U/μL), 2 μL of oligonucleotide primer (10 pmol/μL), 4 μL of dNTPs (2.5 nM), 37.75 μL of distilled water and 1 μL of template containing 1 ng of genomic DNA. To perform Sanger sequencing, purification of PCR products using ethanol precipitation was required prior to the cycle sequencing reaction. Purified PCR products were labelled with the BigDye Terminator Kit v3.1 (Applied Biosystems, USA) for cycle sequencing according to the manufacturer’s procedure. After the second purification of the PCR products, sequencing analysis was performed using the automated sequencer from Applied Biosystems (3730XL DNA Analyzer).

### Statistical analysis

Statistical analyses were performed using SPSS statistics 23.0. Data for continuous variables are presented as the mean ± standard deviation. Continuous variables were analysed using Student’s *t* test. Fisher’s exact test was used to identify differences between categorical variables. The statistical significance was defined as a value of *P* < 0.05 based on two-tailed tests.

## Results

### Variant identification

**Fig. 1:**
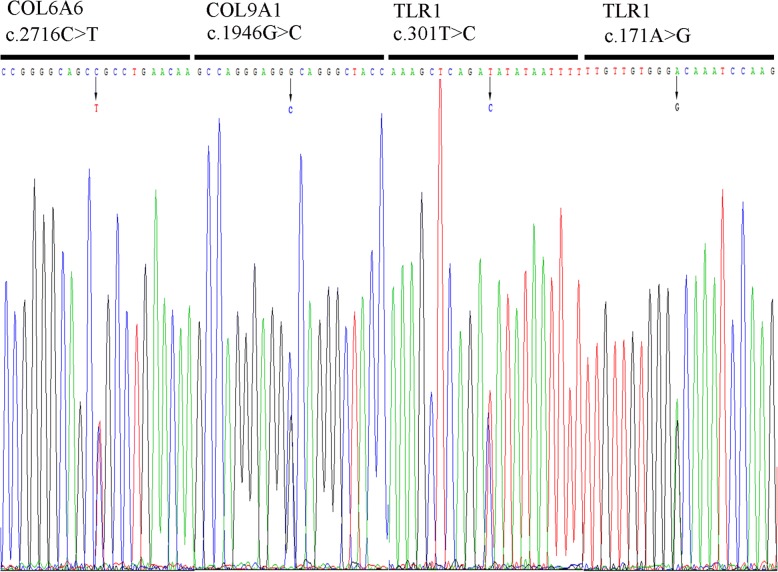
Sanger sequencing chromatograms of four variants in three novel genes. Sanger sequencing confirmed the four variants identified in three novel genes

**Fig. 2:**
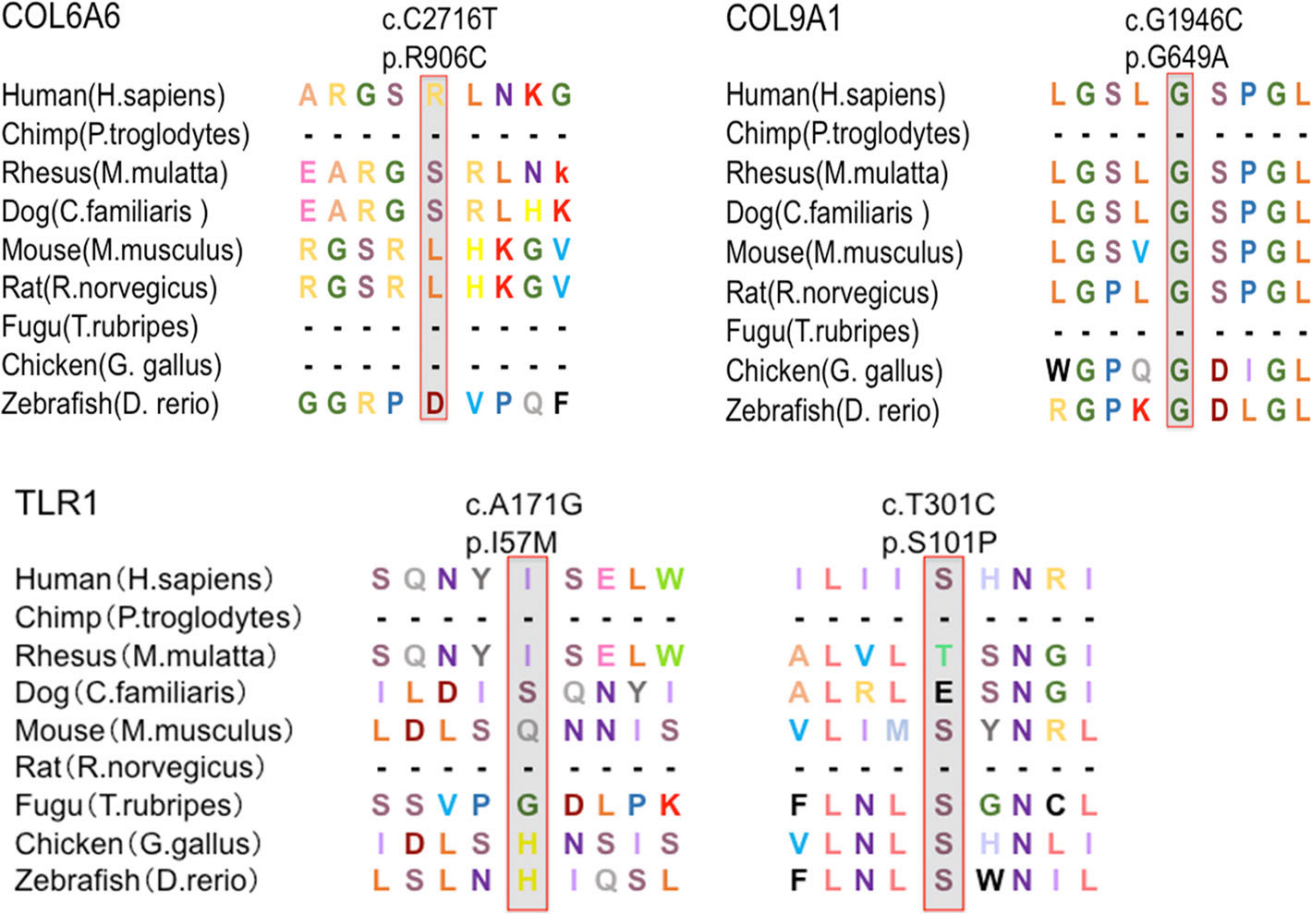
Sequence conservation analysis of four mutations identified in three novel genes in nine species. Sequence conservation analysis was performed for all mutations in nine species. The results indicated that these amino acid residues were evolutionarily conserved in vertebrates

Whole-genome sequencing was performed on 25 DNA samples resulting in an average of 119,525.02 Mb clean reads from the Illumina HiSeq X Ten sequencer. To filter potential pathogenic variants, rare 1000G_EAS≤ 0.005 mutations were identified (based on the BGI database), and damaging variants were predicted by at least four of five algorithms (SIFT, Polyphen2, Mutation Assessor, Mutation Taster and GERP++). As a result, four deleterious variants of three genes were identified in five sporadic patients (Table 1), and these variants were confirmed by Sanger sequencing (Fig. 1). These mutations of three genes (*COL6A6*, *COL9A1* and *TLR1*) accounted for 20.0% of the OPLL patients. The missense mutation of *TLR1* was found in three unrelated patients (accounting for ~ 12.0%). The frequency of mutations in *COL6A1* and *COL9A1* was 4.0% (Table 2). The sequence conservation of the encoded amino acid residues by these SNP mutations was analysed in nine different vertebrate species (Fig. 2). *COL9A1* (c.1946G>C), *TLR1* (c.301 T>C), *TLR1* (c.171A>G) and *COL6A6* (c.2716C>T) were evolutionarily conserved in seven, five, two and one vertebrates, respectively.

**Table 1:** Functional significance of the variants by various algorithms

SNP ID	Gene	Nucleotide change	Protein change	1000G_EAS	SIFT	PolyPhen2	Mutation Assessor	Mutation Taster	GERP++
rs200963433	COL6A6	c.2716C>T	p.Arg906Cys	0.004	D	D	H	D	R
rs201480339	COL9A1	c.1946G>C	p.Gly649Ala	0.001	D	D	H	P	R
rs200212492	TLR1	c.301T>C	p.Ser101Pro	0.001	D	D	H	D	R
rs145135062	TLR1	c.171A>G	p.Ile57Met	0	D	D	M	D	R

1000G_EAS, 1000 Genomes_East Asian; SIFT (D, deleterious); Polyphen2 (D, probably damaging); Mutation Taster (D, disease causing, P, polymorphism automatic); Mutation Assessor (predicted functional, H, high; M, medium); GERP++ (R, rejected_substitutions)

**Table 2:** Clinical information and confirmed variants in all patients

No.	Age	Sex	JOA score	Variants	Gene
1	41	M	6	–	–
2	51	F	8	–	–
3	60	M	6	–	–
4	56	F	6	–	–
5	67	M	1	–	–
6	59	F	8	–	–
7	51	F	4	–	–
8	49	F	3	–	–
9	30	M	3	rs201480339	COL9A1
10	66	M	5	–	–
11	29	M	8	–	–
12	59	F	5	–	–
13	49	F	6	rs145135062	TLR1
14	52	M	6	–	–
15	49	M	9	–	–
16	63	F	5	–	–
17	42	F	5	–	–
18	44	F	5	–	–
19	66	F	8	rs200963433	COL6A6
20	70	M	2	–	–
21	49	F	9	–	–
22	47	M	7	rs200212492	TLR1
23	60	F	2	–	–
24	39	F	10	–	–
25	62	F	6	rs200212492	TLR1

*JOA*, Japanese Orthopedic Association

### Genotype and clinical feature analysis

The genotype/clinical feature correlation was analysed between 5 patients with missense mutations and 20 patients without significant mutations (Table 3). There were no differences between these two groups regarding sex, age and Japanese Orthopaedic Association (JOA) score.

**Table 3:** Clinical features of patients with or without OPLL gene mutations

	Mutation positive (*n* = 5)	Mutation negative (*n* = 20)	*p*
Age	50.8 ± 14.2	52.8 ± 10.5	0.517
Male/female	2/3	8/12	1.000
Thoracic JOA score	6.0 ± 1.9	5.7 ± 2.5	0.316

Data are presented as mean ± SD or *n*; *JOA*, Japanese Orthopedic Association

## Discussion

Several lines of evidence have shown that OPLL is a polygenic disease. Genes encoding proteins and transcription factors involved in osteoblast and chondrocyte development and differentiation have been suggested in the pathomechanism of OPLL. Over the past decade, many sibling-pair linkage studies and candidate gene association studies have reported multiple genes or loci linked to OPLL susceptibility [6, 7, 10–20, 27–34] (Table 4). The majority of previous studies have focused on cervical OPLL rather than T-OPLL. Only one genetic study on T-OPLL has been reported, revealing two deleterious variants of *COL6A1* and *IL17RC* [19]. The present study used whole-genome sequencing to identify candidate pathogenic genes for T-OPLL. The data indicated that the novel variants in three genes (*COL6A6*, *COL9A1* and *TLR1*) may contribute to the pathogenesis of T-OPLL.

**Table 4:** Previously reported genes and microRNAs related to OPLL

Year	First author	Country	Gene	Chromosome	Location
2002	Ogata	Japanese	IL-1β	2q14	Spine
2018	Wang	Chinese	IL17RC	3p25.3 to 3p24.1	Thoracic
1999	Numasawa	Japanese	RXRB	6p21	Spine
1998	Koga	Japanese	COL11A2	6p21	Spine
2010	Liu	Chinese	RUNX2	6p21	Spine
1999	Nakamura	Japanese	NPPS	6q22-q23	Spine
2002	Ogata	Japanese	ESR1	6q25	Spine
2016	Nakajima	Japanese	Rspo2	8q23.1	Spine
2014	Wei	Chinese	PTCH1	9q22.23	Cervical
2011	Kim	Korean	IL15RA	10p15	Spine
2012	Ren	Chinese	BMP9	10q11.22	Cervical
2014	Wei	Chinese	COL17A1	10q24.3-10q25.1	Cervical
2008	Kobashi	Japanese	VDR	12q13	Spine
2006	Horikoshi	Japanese	TGFβ3	14q24	Spine
2002	Furushima	Japanese	BMP4	14q22	Spine
2001	Kamiya	Japanese	TGFβ1	19q13	Cervical
2008	Wang	Chinese	BMP2	20p12.3	Cervical
2003	Tanaka	Japanese	COL6A1	21q22.3	Spine
2018	Wang	Chinese	COL6A1	21q22.3	Thoracic
2014	Chon	Korean	BID	22q11	Cervical
2016	Lim	Korean	miR-146a		Cervical
			miR-149		Cervical
			miR-196a2		Cervical
			miR-499		Cervical
2016	Xu	Chinese	miR-10a-5p		Cervical
			miR-563		Cervical
			miR-885-5p		Cervical
Present	Liang	Chinese	COL6A6	3q22.1	Thoracic
			COL9A1	6q13	Thoracic
			TLR1	4p14	Thoracic

The *COL6A6* gene on chromosome 3q22.1 has 43 exons with a coding region of 6789 bp, and it encodes COL6A6, a 2262-amino acid protein expressed in various tissues, including the muscle, heart and brain. COL6 family proteins are components of the extracellular matrix (ECM) of many tissues, including the bone, cartilage, muscle, tendon and skin. Similar to *COL6A3*, the *COL6A6* gene encodes multiple von Willebrand factor domains and forms a component of the basal lamina of epithelial cells. *COL6A6* may regulate epithelial cell-fibronectin interactions, and variation in this gene may be identified in skin diseases, such as early-onset atopic dermatitis [35]. However, in recent years, *COL6A6* has been implicated as a susceptibility gene in musculoskeletal diseases. Gari et al. performed whole-exome sequencing and showed that COL6A6 mutations can influence OA disease [36]. *COL6A1* has been accepted as a susceptibility gene of OPLL for many years, but there has been no associative study between *COL6A6* and OPLL or T OPLL [14]. *COL6A6* encodes a cell-binding protein, which interacts with COL6A1 protein by forming a trimer in collagen type VI. COL6A1 is involved in membranous or endochondral ossification by providing a scaffold for osteoblastic cells, preosteoblastic cells or chondrocytes. Thus, *COL6A6* mutation may alter the structure and function of collagen type VI and eventually lead to abnormal ossification. The present study is the first to reveal *COL6A6* as a susceptibility gene associated with a predisposition to thoracic OPLL. The function of COL6A6 in the pathogenesis of OPLL will be validated in future studies with respect to cell phenotype and potential signalling pathways.

The *COL9A1* gene is located on chromosome 6q13 and has 41 exons, and it encodes COL9A1, which is involved in synthesizing type IX collagen, a minor collagen component of hyaline cartilage. Previous studies have shown that *COL9A1* is associated with female osteoarthritis (OA), early onset OA and osteoporosis [37–39]. Alfred et al. found that the maturation of cartilage matrix is delayed in COL9A1-deficient mice during bone fracture healing, indicating that endochondral bone formation is impaired during fracture repair in mice [40]. However, the role of COL9A1 in OPLL pathogenesis is unknown. OPLL involves ectopic ossification in the posterior longitudinal ligament, and several histological studies of OPLL have suggested that OPLL develops through a process of endochondral ossification. As COL9A1 is involved in the process of endochondral ossification, it supports the present finding that pathogenic variants in the *COL9A1* gene may be associated with T-OPLL in the Chinese population [41, 42].

The *TLR1* gene is located on chromosome 4p14 and encodes TLR1. TLR1 is a member of the toll-like receptor (TLR) family, which is involved in pathogen recognition and activation of innate immunity. At present, 10 human and 12 murine TLRs have been characterized as TLR1–TLR10 in humans and TLR1–TLR9, TLR11, TLR12 and TLR13 in mice [43]. *TLR1* polymorphism is associated with the susceptibility of multiple diseases, including tuberculosis, pancolitis and prostate cancer [44, 45]. Jeong-Eun et al. found that TLRs can be utilized to induce IL-6 expression in adipose-derived stromal cells (ASCs), and they suggested that the TLR/IL-6 signalling pathway may be useful for modulating osteogenic differentiation of ASCs [46]. In addition, chondrocytes can respond to a wide range of TLR ligands, and TLR1 has been reported to play an important role in the pathogenesis of OA [47]. He Hailong et al. explored the differentially expressed genes (DEGs) in OPLL patient ligament cells and stated that *TLR1* may be involved in spinal cord injury in OPLL [48]. The present study is among the first studies to propose *TLR1* as the disease-causing gene of spine OPLL, especially T-OPLL.

There are crosstalk mechanisms between some of the identified genes and signalling pathways to coordinate osteogenesis and bone homeostasis. Wu et al. found that knockout or mutation of TGF-β and BMP signalling-related genes in mice results in bone abnormalities of underlying osteoblast differentiation and bone formation [49]. Huang et al. determined that TNF-α/IL-1β decreases BMP-2-induced Runx2 expression through the activation of p38 and ERK1/2 signalling to regulate osteoblastic differentiation [50]. However, there is no study concerning the correlations among *COL6A6*, *COL9A1* and *TLR1*. In the future, we will focus on the function of these three novel genes and investigate the relations among them. In addition, fibrosis and calcification are involved in the process of OPLL. In the present study, however, no correlation was noted between these novel genes and fibrosis or calcification.

In the present study, the ratio of missense mutations in *COL6A6*, *COL9A1* and *TLR1* genes was 20% (5/25), which was consistent with previous studies. Wang et al. identified two deleterious mutations in *COL6A1* and *IL17RC* genes in 7 of 30 patients with T-OPLL, yielding a ratio of 23%. Wei et al. proposed that mutations in *PTCH1* and *COL17A1* genes may contribute to the development of cervical OPLL with the ratio of mutations equal to 21% (6/28). Given T-OPLL is a polygenic disease, the definitive prevalence of gene mutations associated with T-OPLL still needs further validation in a large-scale cohort.

The prevalence of OPLL is fairly low compared to degenerative disc diseases, such as lumbar disc herniation and cervical spondylosis. In addition, T-OPLL is less common in comparison with cervical OPLL. To the best of our knowledge, genetic studies on T-OPLL are rare, and the present study is among the first leading studies to investigate pathogenic genes for T-OPLL. Concerning the rare occurrence of T-OPLL, 25 is an adequate number of patients for genetic study. As a next step, the sample size will be increased to reveal additional T-OPLL susceptibility genes. The limitations of the present study were as follows: (1) no replication study was performed to demonstrate the association of the three recently discovered susceptibility loci with T-OPLL; and (2) no functional study was conducted to determine the mechanism and pathways in which the genes involved affect the development of the disease.

In summary, the four identified polymorphisms in three genes may contribute to susceptibility to T-OPLL in the Chinese population. The present study contributed to a better understanding of the aetiology and pathomechanism of T-OPLL. Further replication and functional studies need to be performed to identify pathogenic genes to reduce the incidence and provide novel therapeutic approaches to T-OPLL.

## References

[1] Kawaguchi Y, Nakano M, Yasuda T, Seki S, Hori T, Kimura T (2013). Ossification of the posterior longitudinal ligament in not only the cervical spine, but also other spinal regions: analysis using multidetector computed tomography of the whole spine. Spine.

[2] Fujimori T, Watabe T, Iwamoto Y, Hamada S, Iwasaki M, Oda T (2016). Prevalence, Concomitance, and Distribution of Ossification of the Spinal Ligaments: Results of Whole Spine CT Scans in 1500 Japanese Patients. Spine.

[3] Yang C, Bi Z, Fu C, Zhang Z (2010). A modified decompression surgery for thoracic myelopathy caused by ossification of posterior longitudinal ligament: a case report and literature review. Spine.

[4] Matsumoto M, Chiba K, Toyama Y (2008). Surgical results and related factors for ossification of posterior longitudinal ligament of the thoracic spine: a multi-institutional retrospective study. Spine.

[5] Okamoto K, Kobashi G, Washio M (2004). Dietary habits and risk of ossification of the posterior longitudinal ligaments of the spine (OPLL); findings from a case-control study in Japan. J Bone Miner Metab.

[6] Nakamura I, Ikegawa S, Okawa A (1999). Association of the human NPPS gene with ossification of the posterior longitudinal ligament of the spine (OPLL). Hum Genet.

[7] Horikoshi T, Maeda K, Kawaguchi Y (2006). A large-scale genetic association study of ossification of the posterior longitudinal ligament of the spine. Hum Genet.

[8] Jekarl DW, Paek CM, An YJ (2013). TGFBR2 gene polymorphism is associated with ossification of the posterior longitudinal ligament. J Clin Neurosci.

[9] Jun JK, Kim SM (2012). Association study of fibroblast growth factor 2 and fibroblast growth factor receptors gene polymorphism in korean ossification of the posterior longitudinal ligament patients. J Korean Neurosurg Soc.

[10] Kamiya M, Harada A, Mizuno M, Iwata H, Yamada Y (2001). Association between a polymorphism of the transforming growth factor-beta1 gene and genetic susceptibility to ossification of the posterior longitudinal ligament in Japanese patients. Spine.

[11] Numasawa T, Koga H, Ueyama K (1999). Human retinoic X receptor beta: complete genomic sequence and mutation search for ossification of posterior longitudinal ligament of the spine. J Bone Miner Res.

[12] Ogata N, Koshizuka Y, Miura T (2002). Association of bone metabolism regulatory factor gene polymorphisms with susceptibility to ossification of the posterior longitudinal ligament of the spine and its severity. Spine.

[13] Ren Y, Liu ZZ, Feng J (2012). Association of a BMP9 haplotype with ossification of the posterior longitudinal ligament (OPLL) in a Chinese population. PLoS One.

[14] Tanaka T, Ikari K, Furushima K (2003). Genomewide linkage and linkage disequilibrium analyses identify COL6A1, on chromosome 21, as the locus for ossification of the posterior longitudinal ligament of the spine. Am J Hum Genet.

[15] Wang H, Liu D, Yang Z (2008). Association of bone morphogenetic protein-2 gene polymorphisms with susceptibility to ossification of the posterior longitudinal ligament of the spine and its severity in Chinese patients. Eur Spine J.

[16] Kim DH, Jeong YS, Chon J (2011). Association between interleukin 15 receptor, alpha (IL15RA) polymorphism and Korean patients with ossification of the posterior longitudinal ligament. Cytokine.

[17] Liu Y, Zhao Y, Chen Y, Shi G, Yuan W (2010). RUNX2 polymorphisms associated with OPLL and OLF in the Han population. Clin Orthop Relat Res.

[18] Nakajima M, Kou I, Ohashi H, Ikegawa S (2016). Identification and Functional Characterization of RSPO2 as a Susceptibility Gene for Ossification of the Posterior Longitudinal Ligament of the Spine. Am J Hum Genet.

[19] Wang P, Liu X, Zhu B (2018). Identification of susceptibility loci for thoracic ossification of the posterior longitudinal ligament by whole-genome sequencing. Mol Med Rep.

[20] Nakajima M, Takahashi A, Tsuji T (2014). A genome-wide association study identifies susceptibility loci for ossification of the posterior longitudinal ligament of the spine. Nat Genet.

[21] Belkadi A, Bolze A, Itan Y (2015). Whole-genome sequencing is more powerful than whole-exome sequencing for detecting exome variants. Proc Natl Acad Sci U S A.

[22] Chen X, Guo J, Cai T (2016). Targeted next-generation sequencing reveals multiple deleterious variants in OPLL-associated genes. Sci Rep.

[23] Adzhubei IA, Schmidt S, Peshkin L (2010). A method and server for predicting damaging missense mutations. Nat Methods.

[24] Sim NL, Kumar P, Hu J, Henikoff S, Schneider G, Ng PC (2012). SIFT web server: predicting effects of amino acid substitutions on proteins. Nucleic Acids Res.

[25] Schwarz JM, Cooper DN, Schuelke M, Seelow D (2014). MutationTaster2: mutation prediction for the deep-sequencing age. Nat Methods.

[26] Davydov EV, Goode DL, Sirota M, Cooper GM, Sidow A, Batzoglou S (2010). Identifying a high fraction of the human genome to be under selective constraint using GERP++. PLoS Comput Biol.

[27] Stapleton CJ, Pham MH, Attenello FJ, Hsieh PC (2011). Ossification of the posterior longitudinal ligament: genetics and pathophysiology. Neurosurg Focus.

[28] Koga H, Sakou T, Taketomi E (1998). Genetic mapping of ossification of the posterior longitudinal ligament of the spine. Am J Hum Genet.

[29] Wei W, He HL, Chen CY (2014). Whole exome sequencing implicates PTCH1 and COL17A1 genes in ossification of the posterior longitudinal ligament of the cervical spine in Chinese patients. Genet Mol Res.

[30] Kobashi G, Ohta K, Washio M (2008). FokI variant of vitamin D receptor gene and factors related to atherosclerosis associated with ossification of the posterior longitudinal ligament of the spine: a multi-hospital case-control study. Spine.

[31] Furushima K, Shimo-Onoda K, Maeda S (2002). Large-scale screening for candidate genes of ossification of the posterior longitudinal ligament of the spine. J Bone Miner Res.

[32] Chon J, Hong JH, Kim J (2014). Association between BH3 interacting domain death agonist (BID) gene polymorphism and ossification of the posterior longitudinal ligament in Korean population. Mol Biol Rep.

[33] Lim JJ, Shin DA, Jeon YJ (2016). Association of miR-146a, miR-149, miR-196a2, and miR-499 Polymorphisms with Ossification of the Posterior Longitudinal Ligament of the Cervical Spine. PLoS One.

[34] Xu C, Chen Y, Zhang H (2016). Integrated microRNA-mRNA analyses reveal OPLL specific microRNA regulatory network using high-throughput sequencing. Sci Rep.

[35] Heo WI, Park KY, Jin T (2017). Identification of novel candidate variants including COL6A6 polymorphisms in early-onset atopic dermatitis using whole-exome sequencing. BMC Med Genet.

[36] Gari MA, AlKaff M, Alsehli HS (2016). Identification of novel genetic variations affecting osteoarthritis patients. BMC Med Genet.

[37] Snelgrove TA, Peddle LJ, Stone C (2005). Association of COL1A2, COL2A1 and COL9A1 and primary osteoarthritis in a founder population. Clin Genet.

[38] Mustafa Z, Chapman K, Irven C (2000). Linkage analysis of candidate genes as susceptibility loci for osteoarthritis-suggestive linkage of COL9A1 to female hip osteoarthritis. Rheumatology (Oxford, England).

[39] Wang CJ, Iida K, Egusa H, Hokugo A, Jewett A, Nishimura I (2008). Trabecular bone deterioration in col9a1+/- mice associated with enlarged osteoclasts adhered to collagen IX-deficient bone. J Bone Miner Res.

[40] Opolka A, Ratzinger S, Schubert T (2007). Collagen IX is indispensable for timely maturation of cartilage during fracture repair in mice. Matrix biology.

[41] Dreier R, Opolka A, Grifka J, Bruckner P, Grassel S (2008). Collagen IX-deficiency seriously compromises growth cartilage development in mice. Matrix biology.

[42] Liu H, Zhao Z, Clarke RB, Gao J, Garrett IR, Margerrison EE (2013). Enhanced tissue regeneration potential of juvenile articular cartilage. Am J Sports Med.

[43] Lee CC, Avalos AM, Ploegh HL (2012). Accessory molecules for Toll-like receptors and their function. Nat Rev Immunol.

[44] Noreen M, Arshad M (2015). Association of TLR1, TLR2, TLR4, TLR6, and TIRAP polymorphisms with disease susceptibility. Immunol Res.

[45] Qi H, Sun L, Wu X (2015). Toll-like receptor 1(TLR1) Gene SNP rs5743618 is associated with increased risk for tuberculosis in Han Chinese children. Tuberculosis (Edinburgh, Scotland).

[46] Huh JE, Lee SY (2013). IL-6 is produced by adipose-derived stromal cells and promotes osteogenesis. Biochim Biophys Acta.

[47] Sillat T, Barreto G, Clarijs P (2013). Toll-like receptors in human chondrocytes and osteoarthritic cartilage. Acta Orthop.

[48] He H, Mao L, Xu P (2014). Ossification of the posterior longitudinal ligament related genes identification using microarray gene expression profiling and bioinformatics analysis. Gene.

[49] Wu M, Chen G, Li YP (2016). TGF-beta and BMP signaling in osteoblast, skeletal development, and bone formation, homeostasis and disease. Bone research.

[50] Huang RL, Yuan Y, Tu J, Zou GM, Li Q (2014). Opposing TNF-alpha/IL-1beta- and BMP-2-activated MAPK signaling pathways converge on Runx2 to regulate BMP-2-induced osteoblastic differentiation. Cell Death Dis.

